# Complete Genome Sequence of *Bacillus velezensis* L-1, Which Has Antagonistic Activity against Pear Diseases

**DOI:** 10.1128/genomeA.01271-17

**Published:** 2017-11-30

**Authors:** Pingping Sun, Jianchao Cui, Xiaohui Jia, Wenhui Wang

**Affiliations:** Institute of Pomology, Chinese Academy of Agricultural Sciences, Xingcheng, Liaoning, China

## Abstract

*Bacillus velezensis* L-1 is an effective biocontrol agent against pear diseases. Here, we report the complete genome sequence of *B. velezensis* L-1 in which clusters related to the biosynthesis of secondary metabolites were predicted. This genome provides insights into the possible biocontrol mechanisms and furthers application of this specific bacterium.

## GENOME ANNOUNCEMENT

*Bacillus velezensis* L-1 was originally isolated from rhizosphere soil of the China Pear Germplasm Repository located in Xingcheng, Liaoning, China. It was selected based on its outstanding antagonistic activity against *Botryosphaeria berengeriana* (the causing agent of pear ring rot) and wide antagonistic spectrum against pear pathogens (1). Previous work found that *B. velezensis* L-1 colonized efficiently in pear wounds, caused abnormal growth of the *B. berengeriana* mycelium, stimulated the expression of defense-related enzymes in pears, and maintained the quality of the fruit (1). *B. velezensis* L-1 is an effective biocontrol agent against pear diseases, but its biocontrol mechanisms are currently unclear. The whole-genome sequence of L-1 will further our ability to both understand its biocontrol mechanisms and facilitate its application.

The complete genome of strain L-1 was sequenced with the PacBio RSII platform at Biomarker Technologies Co., Ltd. (Beijing, China). The obtained data were assembled *de novo* using the Hierarchical Genome Assembly Process (HGAP version 3.0) and annotated with Prodigal version 2.50 (2), tandem repeats finder software (3), tRNAscan-SE (4), and RNAmmer (5). Their functional classifications were determined by aligning these sequences to the Clusters of Orthologous Groups (COG) (6), KEGG (7), and Gene Ontology (GO) databases (8). The carbohydrate-active enzyme analyses were performed by downloading the Carbohydrate-Active Enzymes database (9) from dbCan (http://csbl.bmb.uga.edu/dbCAN) and annotating with HMMER software (10). Prophages were predicted with PHAST (11), and the secondary metabolites were predicted by antiSMASH version 3.0 (12).

The *B. velezensis* L-1 genome comprises a 4,090,582-bp chromosome with a GC content of 46.52%. About 3,624,780 bp of the nucleotide sequences were predicted to have 3,978 coding sequences, 44 pseudogenes, 126 repeated genes, 86 tRNAs, and 27 rRNAs. Approximately 73.61% of the genes were assigned to specific COGs, and 51.45% were involved in predicted metabolic pathways. We found that 459 genes were related to carbohydrate enzymes, including 40 genes involved in carbohydrate-binding modules, 75 genes coding carbohydrate esterases, 118 genes coding glycoside hydrolases, 183 genes coding glycosyl transferases, and 6 and 37 genes related to polysaccharide lyases and auxiliary activities, respectively. Four prophages with a total of 210.5 kb were predicted. We found that several genes were related to the biocontrol pathways in strain L-1, such as the production of secondary antibiotics, including bacillibactin, teichuronic acid, bacilysin, macolactin, bacillaene, fengycin, difficidin, surfactin, and plantazolicin, providing the basis for its biocontrol phenomenon.

### Accession number(s).

The strain was stored in the China General Microbiological Culture Collection Center under the accession number CGMCC14373. The complete genome sequence of *Bacillus velezensis* L-1 has been deposited in GenBank under the GenBank accession number CP023859.
